# Field sources in a Lorentz-symmetry breaking scenario with a single background vector

**DOI:** 10.1140/epjc/s10052-014-2937-9

**Published:** 2014-06-25

**Authors:** L. H. C. Borges, F. A. Barone, J. A. Helayël-Neto

**Affiliations:** 1 Universidade Federal do ABC, Centro de Ciências Naturais e Humanas, Rua Santa Adélia, 166, Santo André, SP 09210-170 Brazil; 2 IFQ, Universidade Federal de Itajubá, Av. BPS 1303, Pinheirinho, Caixa Postal 50, Itajubá, MG 37500-903 Brazil; 3 Centro Brasileiro de Pesquisas Físicas, Rua Dr. Xavier Sigaud 150, Urca, Rio de Janeiro, RJ 22290-180 Brazil

## Abstract

This paper is devoted to an investigation of the interactions between stationary sources of the electromagnetic field, in a model which exhibits explicit Lorentz-symmetry breaking due to the presence of a single background vector. We focus on physical phenomena that emerge from this kind of breaking and which have no counterpart in Maxwell electrodynamics.

## Introduction

Since the proposal of Carroll et al. in [1], a great deal of effort has been devoted to the study of models which exhibit explicit Lorentz-symmetry breaking in many contexts. Among them, models stand out where the Lorentz-symmetry is broken in the gauge field sector. We mention, for instance, the study of classical electrodynamics with Lorentz-symmetry violation [2–6], QED effects [7–15], electromagnetic wave propagation [9, 11–13, 16–21], the extension of the Standard Model for Lorentz-symmetry breaking scenarios [22, 23], non-minimal couplings [24–26], extensions of Lorentz-symmetry breaking with derivatives of higher order [27], the Casimir effect [28], classical point-like particles dynamics [29], connections between axion physics and Lorentz-symmetry breaking models [14, 15, 30], and so on.

Regarding the Abelian gauge fields, we have two kinds of models which exhibit Lorentz-symmetry breaking: the tensorial model [2] and the Carroll–Field–Jackiw one [1], which is a generalization of the Chern–Simons model for $(3+1)$ dimensions. The former is richer in comparison with the latter, once it has more parameters, and thus may lead to more physical effects with no counterpart in Lorentz-invariant theories.

One of the most fundamental questions that can be asked in any gauge theory concerns the interaction between external field sources. This subject has been considered in the context of Lorentz-symmetry breaking theories [2], but some points are still worth considering, not only for their theoretical aspects, but also because of their possible experimental relevance in the search for physical phenomena with no counterparts in Maxwell theory.

This paper is devoted to the investigation of electromagnetic phenomena in a Lorentz-symmetry breaking scenario, with no counterpart in Maxwell theory. We consider a tensorial model and restrict our analysis to the non-birefringent sector [2], parameterizing the background tensor in such a way as to reduce it to one background vector, which allows us to obtain exact results. We also focus attention on the case of stationary sources.

Specifically, we obtain exactly the photon propagator of the model in Sect. 2. In Sect. 3 we consider effects due to the presence of point-like stationary charges. In Sect. 4 we compute the interaction energy between a steady line current and a point-like stationary charge. Section 5 is devoted to the study of physical phenomena due to the presence of one or two Dirac strings, and Sect. 6 is devoted to our final remarks and conclusions.

## The model

In this paper let us consider the electromagnetic field in a Lorentz-symmetry breaking scenario parametrized by just one background vector. The Lorentz-symmetry breaking is accomplished by a tensorial coupling of the electromagnetic field in the Lagrangian, as follows:

1 $$\begin{aligned} \mathcal{L}&= -\frac{1}{4}F_{\mu \nu }F^{\mu \nu }\!-\!\frac{1}{2\gamma }\left( \partial _{\mu }A^{\mu }\right) ^{2}\!-\!\frac{1}{2}v^ {\mu }v_{\nu }F_{\mu \lambda }F^{\nu \lambda }\!+\!J^{\mu }A_{\mu },\nonumber \\ \end{aligned}$$

where $A^{\mu }$ is the electromagnetic field, $F^{\mu \nu }=\partial ^{\mu }A^{\nu }-\partial ^{\nu }A^{\mu }$ is the field strength, $J^{\mu }$ is the external source, $\gamma$ is a gauge parameter, and $v^\mu$ is the background vector. We will be working in a $3+1$ dimensional space-time with Minkowski metric $\eta ^{\mu \nu }=(+,-,-,-)$.

As the Lorentz-symmetry breaking must be very tiny, the vector $v^{\mu }$, which is a dimensionless quantity, must be small.

In reference [26], two points are discussed in connection with the model (1). The first one is the fact that the Lagrangian (1) can be obtained from a model with a non-minimal coupling of the electromagnetic field with the spinorial one. The second point relies on the fact that Eq. (1) is a special case of the Lagrangian proposed in [2], where we have a tensorial parameter controlling the Lorentz-symmetry breaking with a term $\sim k_{\mu \nu \alpha \beta }F^{\alpha \beta }F^{\mu \nu }$. That is, when $k_{\mu \nu \alpha \beta } \sim \eta _{\mu \alpha }v_{\nu }v_{\beta }- \eta _{\nu \alpha }v_{\mu }v_{\beta }+\eta _{\nu \beta }v_{\mu }v_{\alpha }-\eta _{\mu \beta }v_{\nu }v_{\alpha }$ the model discussed in [2] is given by Eq. (1).

The propagator $D^{\mu \nu }(x,y)$ satisfies the differentialequation

2 $$\begin{aligned}&\Biggl [\left( \partial ^{2}+(v\cdot \partial )^{2}\right) \eta ^{\mu \nu }-\left( 1-\frac{1}{\gamma }\right) \partial ^{\mu }\partial ^{\nu }+v^{\mu }v^{\nu }\partial ^{2}\nonumber \\&\qquad -\left( v\cdot \partial \right) v^{\mu }\partial ^{\nu }-\left( v\cdot \partial \right) v^{\nu }\partial ^{\mu }\Biggr ]{D_{\nu }}^{\lambda }\left( x,y\right) \nonumber \\&\quad =\eta ^{\mu \lambda }\delta ^{d+D+1}\left( x-y\right) , \end{aligned}$$

where $$\partial ^{2}=\partial ^{\mu }\partial _{\mu }$$ and $$v\cdot \partial =v^{\mu }\partial _{\mu }$$.

Finding the Fourier transform and taking the Lorentz gauge condition, with $\gamma =1$, one readily shows that the propagator is given by

3 $$\begin{aligned}&D^{\mu \nu }(x,y)=-\int \frac{\mathrm{d}^{4}p}{(2\pi )^{4}}\frac{e^{-ip\cdot (x-y)}}{[p^{2}+(p\cdot v)^2]}\Bigg [\eta ^{\mu \nu }-\frac{v^{\mu }v^{\nu }}{1+v^{2}}\nonumber \\&\quad -\frac{v^{2}}{1+v^{2}}\frac{(p\cdot v)^2}{p^{2}}\frac{p^{\mu }p^{\nu }}{p^{2}}\!+\!\frac{1}{1+v^{2}}\frac{(p\cdot v)}{p^{2}}(p^{\mu }v^{\nu }\!+\!v^{\mu }p^{\nu })\Bigg ].\nonumber \\ \end{aligned}$$

## Point-like charges

In this section we study the interaction energy between two stationary point-like field sources in $(3+1)$ dimensions. The results can easily be extended to an arbitrary number of spatial dimensions.

In the first model, we consider the field sources described by the time-independent current

4 $$\begin{aligned} J_{\mu }^{I}(\mathbf{x})=\sigma _{1}\eta ^{0}_{\mu }\delta ^{3}\left( \mathbf{x}-\mathbf{a}_ {1}\right) +\sigma _{2}\eta ^{0}_{\mu }\delta ^{3}\left( \mathbf{x}-\mathbf{a}_ {2}\right) \!, \end{aligned}$$

where we have two spatial Dirac delta functions, concentrated at the positions $\mathbf{a}_{1}$ and $\mathbf{a}_{2}$. The parameters $\sigma _{1}$ and $\sigma _{2}$ are the coupling constants between the field and the delta functions, and they can be seen as electric charges.

Once we have a quadratic Lagrangian in the field variables, as discussed in references [31–33], the contribution due to the sources to the ground state energy of the system is given by

5 $$\begin{aligned} E=\frac{1}{2T}\int \int \mathrm{d}^{4}x\ \mathrm{d}^{4}y J^{\mu }(x)D_{\mu \nu }(x,y)J^{\nu }(x), \end{aligned}$$

where $T$ is the time variable.

Substituting Eqs. (3) and (4) into Eq. (5), discarding the self-interacting contributions (that is, the interactions of a given point charge with itself), computing the integrals in the following order: $\mathrm{d}^{3}\mathbf{x}$, $\mathrm{d}^{3}\mathbf{y}$, $\mathrm{d}x^{0}$, $\mathrm{d}p^{0}$, and $\mathrm{d}y^{0}$, using the Fourier representation for the Dirac delta function $\delta (p^{0})=\int \mathrm{d}x/(2\pi )\exp (-ipx)$, and identifying the time interval as $T=\int \mathrm{d}y^{0}$, we write

6 $$\begin{aligned} E^{I}=\sigma _{1}\sigma _{2}\left( 1-\frac{(v^{0})^{2}}{1+v^{2}}\right) \int \frac{\mathrm{d}^{3}\mathbf{p}}{(2\pi )^{3}}\frac{\exp (i\mathbf{p}\cdot \mathbf{a})}{[\mathbf{p}^2-(\mathbf{v}\cdot \mathbf{p})^{2}]}, \end{aligned}$$

where we have defined $\mathbf {a}=\mathbf{a}_{1}-\mathbf{a}_{2}$, which is the distance between the two electric charges.

In order to calculate the integral in Eq. (6), we first split the vector $\mathbf p$ into two parts, one parallel, $\mathbf{p}_{p}$, and the other normal, $\mathbf{p}_{n}$, to the vector $\mathbf v$, namely

7 $$\begin{aligned} \mathbf{p}=\mathbf{p}_{n}+\mathbf{p}_{p},\ \ \mathbf{p}_{p}=\mathbf{v}\Bigl (\frac{\mathbf{v}\cdot \mathbf{p}}{\mathbf{v}^{2}}\Bigr ),\ \ \mathbf{p}_{n}=\mathbf{p}-\mathbf{v}\Bigl (\frac{\mathbf{v}\cdot \mathbf{p}}{\mathbf{v}^{2}}\Bigr ), \end{aligned}$$

where $\mathbf{p}_{n}\cdot \mathbf{v}=0$. Now we define the vector $\mathbf{q}$ by

8 $$\begin{aligned} \mathbf{q}=\mathbf{p}_{n}+\mathbf{p}_{p}\sqrt{1-\mathbf{v}^{2}}=\mathbf{p}+\mathbf{v}\Bigl (\frac{\mathbf{v}\cdot \mathbf{p}}{\mathbf{v}^{2}}\Bigr )(\sqrt{1-\mathbf{v}^{2}}-1). \end{aligned}$$

With the definitions (7) and (8) one may write

9 $$\begin{aligned}&\mathbf{p}_{p}=\frac{{\mathbf {v}} ({\mathbf {v}}\cdot {\mathbf {q}})}{\mathbf{v}^{2}\sqrt{1-\mathbf{v}^{2}}} ,\quad \mathbf{p}_{n}={\mathbf {q}}-\frac{{\mathbf {v}}({\mathbf {v}}\cdot {\mathbf {q}})}{\mathbf{v}^{2}}\nonumber \\&\quad \Rightarrow \mathbf{p}=\mathbf{q}+\frac{({{\mathbf {v}}}\cdot \mathbf{q}){{\mathbf {v}}}}{\mathbf{v}^{2}}\left( \frac{1}{\sqrt{1-\mathbf{v}^{2}}}-1\right) ,\end{aligned}$$

10 $$\begin{aligned}&\mathbf{q}^{2}=\mathbf{p}^2-(\mathbf{v}\cdot \mathbf{p})^{2}. \end{aligned}$$

With the aid of the definition

11 $$\begin{aligned} \mathbf{b}=\mathbf{a}+\frac{1-\sqrt{1-\mathbf{v}^{2}}}{\sqrt{1-\mathbf{v}^{2}}}\frac{\mathbf{v}\cdot \mathbf{a}}{\mathbf{v}^{2}}\mathbf{v}\ \end{aligned}$$

and Eq. (9), we also have

12 $$\begin{aligned} \mathbf{p}\cdot \mathbf{a}=\mathbf{b}\cdot \mathbf{q}. \end{aligned}$$

The Jacobian of the transformation from $\mathbf{p}$ to $\mathbf{q}$ can be calculated from Eq. (9),

13 $$\begin{aligned} \det \left[ \frac{\partial \mathbf {p}}{\partial \mathbf {q}}\right] =\frac{1}{\sqrt{1-\mathbf{v}^{2}}}. \end{aligned}$$

Substituting Eqs. (10) and (12) into Eq. (6) and using Eq. (13) we have

14 $$\begin{aligned} E^{I}=\frac{\sigma _{1}\sigma _{2}}{\sqrt{1-\mathbf{v}^{2}}}\left( 1-\frac{(v^{0})^{2}}{1+v^{2}}\right) \int \frac{\mathrm{d}^{3}\mathbf{q}}{(2\pi )^{3}}\frac{\exp (i\mathbf{b}\cdot \mathbf{q})}{\mathbf{q}^2}. \end{aligned}$$

Using the fact that [32]

15 $$\begin{aligned} \int \frac{\mathrm{d}^{3}\mathbf{q}}{(2\pi )^{3}}\frac{\exp (i\mathbf{b}\cdot \mathbf{q})}{\mathbf{q}^2}=\frac{1}{4\pi |\mathbf{b}|} \end{aligned}$$

and Eq. (11), and performing some simple manipulations we have, finally,

16 $$\begin{aligned} E^{I}=\frac{\sigma _{1}\sigma _{2}}{4\pi }\frac{\sqrt{1-\mathbf{v}^{2}}}{1+v^2}\left[ \mathbf{a}^{2}+\frac{(\mathbf{v}\cdot \mathbf{a})^{2}}{1-\mathbf{v}^{2}}\right] ^{-1/2}. \end{aligned}$$

Equation (16) is an exact result and gives the interaction energy between two point-like charges for the model (1). If we take $v^{\mu }=0$, the expression (16) reduces to the well-known Coulomb interaction. In Eq. (16) the coefficient $\sqrt{1-\mathbf{v}^{2}}/(1+v^2)$ can be absorbed into the definition of the electric charges $\sigma _{1}$ and $\sigma _{2}$ and does not indicate a Lorentz-symmetry breaking. The extra factor proportional to $(\mathbf{v}\cdot \mathbf{a})^{2}$ inside the brackets is an evident contribution which evinces the Lorentz-symmetry breaking. It leads to an anisotropic interaction between the charges. This fact can be clarified if we compute the force on the charge $2$; for instance,

17 $$\begin{aligned} \mathbf{F}^{I}=-\nabla E^{I}=\frac{\sigma _{1}\sigma _{2}}{4\pi }\frac{1-\mathbf{v}^{2}}{1+v^{2}}\frac{1}{\mathbf{a}^{2}}\frac{(1-\mathbf{v}^{2}){\hat{a}}+(\mathbf{v}\cdot {\hat{a}})\mathbf{v}}{\left[ 1-\mathbf{v}^{2}+(\mathbf{v}\cdot {\hat{a}})^{2}\right] ^{3/2}}.\nonumber \\ \end{aligned}$$

Notice that Eq. (17) is a long-range but anisotropic interaction. In the special situation where $\mathbf{v}$ and ${\hat{a}}$ are perpendicular to each other, the force (17) becomes a Coulomb interaction with effective charges $\sigma \rightarrow \sigma \sqrt{\frac{(1-\mathbf{v}^{2})^{1/2}}{1+v^{2}}}$. In the special case where $\mathbf{v}=0$ the force (17) still exhibits a Coulomb-like behavior, with electric charges renormalized by $\sigma \rightarrow \sigma [1+(v^{0})^{2}]^{-1/2}$.

Since $v^{\mu }$ is a small quantity, it is relevant to expand the expression (17) in lowest order in $v^{\mu }$,

18 $$\begin{aligned} \mathbf{F}^{I}&\cong \frac{\sigma _{1}\sigma _{2}}{4\pi }\frac{1}{\mathbf{a}^{2}}\Biggl [\Biggl (1-(v^{0})^{2}+\frac{1}{2}\mathbf{v}^{2}\Biggr ){\hat{a}}\nonumber \\&+(\mathbf{v}\cdot {\hat{a}})\Biggl (\mathbf{v}-\frac{3}{2}(\mathbf{v}\cdot {\hat{a}}){\hat{a}}\Biggr )\Biggr ]. \end{aligned}$$

The first contribution inside the brackets in Eq. (18), proportional to ${\hat{a}}$, exhibits a typical inverse-square radial law. The second contribution inside the brackets is a long-range one, but it exhibits anisotropy.

An important consequence of the anisotropies in expression (16) is the emergence of a spontaneous torque on an electric dipole. In order to investigate this effect, let us consider a typical dipole composed by two opposite electric charges placed at a fixed distance apart. For this task we use the expression (17) with $\sigma _{1}=-\sigma _{2}=\sigma$, $\mathbf{a}_{1}=\mathbf{R}+\frac{\mathbf{A}}{2}$, and $\mathbf{a}_{2}=\mathbf{R}-\frac{\mathbf{A}}{2}$, where we take the distance vector $\mathbf A$ fixed (and small). Thus, the interaction energy between two charges (16) becomes

19 $$\begin{aligned} E_{\mathrm{dipole}}=-\frac{\sigma ^{2}}{4\pi A}\frac{1-\mathbf{v}^{2}}{1+v^{2}}\left( 1-\mathbf{v}^{2}\sin ^{2}\theta \right) ^{-1/2}, \end{aligned}$$

where $A=\left| \mathbf A\right|$ and $\theta$ is the angle between the vectors $\mathbf{A}$ and $\mathbf v$. Notice that $0\le \theta \le \pi$. The energy (19) leads to a spontaneous torque on the dipole, as follows:

20 $$\begin{aligned} \tau _{\mathrm{dipole}}&= -\frac{\partial E}{\partial \theta }=\frac{\sigma ^{2}}{8\pi A}\frac{1-\mathbf{v}^{2}}{1+v^{2}}\frac{\mathbf{v}^{2}\sin (2\theta )}{\left( 1-\mathbf{v}^{2}\sin ^{2}\theta \right) ^{3/2}}\nonumber \\&\cong \frac{\sigma ^{2}\mathbf{v}^{2}}{8\pi A}\sin (2\theta ). \end{aligned}$$

Equation (20) shows that the torque on a dipole is an exclusive effect due to the Lorentz-symmetry breaking. For $\theta =0,\pi /2,\pi$ the torque vanishes. When $\theta =\pi /4,$ the torque exhibits a maximum value.

It is worth mentioning that the results above can be naturally generalized to an arbitrary number of spatial dimensions, as in [32, 33].

## A steady current line and a point-like charge

In this section we study the interaction energy between a steady line current and a point-like stationary charge, the second model studied in this paper. The steady current line shall be taken to flow parallel to the $z$-axis, along the straight line located at $\mathbf{A}=(A^{1},A^{2},0)$. The electric charge is placed at position $\mathbf{a}_{(2)}$. The whole external source of the system is given by

21 $$\begin{aligned} J_{\mu }^{II}\left( x\right) =\sigma _{1}\eta ^{3}_{\ \mu }\delta ^{2}\left( \mathbf{x}_{\perp }-\mathbf{A}\right) +\sigma _{2}\eta ^{0}_{\ \mu }\delta ^{3}\left( \mathbf{x}-\mathbf{a}_{(2)}\right) , \end{aligned}$$

where $\mathbf{x}_{\perp }=(x^{1},x^{2},0)$ is the position vector perpendicular to the straight line current. The parameters $\sigma _{1}$ and $\sigma _{2}$ stand for the current intensity and electric charge strength, respectively.

Substituting Eq. (21) into Eq. (5), discarding self-interacting terms and using the fact that $D^{30}(x,y)=D^{03}(x,y)$, which can be seen from Eq. (3), one shows that

22 $$\begin{aligned} E^{II}&= \frac{\sigma _{1}\sigma _{2}}{T}\int \int \mathrm{d}^{4}x\ \mathrm{d}^{4}y\ D^{30}(x,y)\nonumber \\&\times \delta ^{2}\left( \mathbf{x}_{\perp }-\mathbf{A}\right) \delta ^{3}\left( \mathbf{y}-\mathbf{a}_{(2)}\right) . \end{aligned}$$

Substituting the propagator (3) in Eq. (5), performing (in this order) the integrals $\mathrm{d}^{2}\mathbf{x}_{\perp }$, $\mathrm{d}^{3}\mathbf{y}$, $\mathrm{d}x^{3}$, $\mathrm{d}p^{3}$, $\mathrm{d}x^{0}$, $\mathrm{d}p^{0}$, and $\mathrm{d}y^{0}$, and identifying the time interval $\int \mathrm{d}y^{0}=T$, we have

23 $$\begin{aligned} E^{II}=-\sigma _{1}\sigma _{2}\left( \frac{v^{3}v^{0}}{1+v^{2}}\right) \int \frac{\mathrm{d}^{2}\mathbf{p}_{\perp }}{(2\pi )^{2}}\frac{\exp (i\mathbf{p}_{\perp }\cdot \mathbf{a}_{\perp })}{[\mathbf{p}_{\perp }^2-(\mathbf{v}_{\perp }\cdot \mathbf{p}_{\perp })^{2}]},\nonumber \\ \end{aligned}$$

where we defined $\mathbf{p}_{\perp }=(p^{1},p^{2},0)$ and the distance between the charge and the line current $\mathbf{a}_{\perp }=\mathbf{A}-\mathbf{a}_{(2)}=(A^{1}-a^{1}_{(2)},A^{2}-a^{2}_{(2)},0)$.

In order to solve the integral above we employ the same method as used in the previous section, with the transformations (7), (8), and (11) carried out only for the perpendicular components to the straight line current of the vector involved,

24 $$\begin{aligned} E^{II}\!=\!-\!\frac{\sigma _{1}\sigma _{2}}{\sqrt{1\!-\!\mathbf{v}_{\perp }^{2}}}\left( \frac{v^{3}v^{0}}{1\!+\!v^{2}}\right) \int \frac{\mathrm{d}^{2}\mathbf{q}_{\perp }}{(2\pi )^{2}}\frac{\exp (i\mathbf{q}_{\perp }\cdot \mathbf{b}_{\perp })}{\mathbf{q}_{\perp }^2}.\quad \end{aligned}$$

The integral in Eq. (24) is divergent. In order to circumvent this problem we proceed as in reference [32], introducing a mass regulator parameter, as follows:

25 $$\begin{aligned} E^{II}=-\frac{\sigma _{1}\sigma _{2}}{\sqrt{1-\mathbf{v}_{\perp }^{2}}}\left( \frac{v^{3}v^{0}}{1+v^{2}}\right) \nonumber \\ \lim _{m\rightarrow 0}\int \frac{\mathrm{d}^{2}\mathbf{q}_{\perp }}{(2\pi )^{2}}\frac{\exp (i\mathbf{q}_{\perp }\cdot \mathbf{b}_{\perp })}{\mathbf{q}_{\perp }^2+m^{2}}, \end{aligned}$$

and using the fact that [32]

26 $$\begin{aligned} \int \frac{\mathrm{d}^{2}\mathbf{q}_{\perp }}{(2\pi )^{2}}\frac{\exp (i\mathbf{q}_{\perp }\cdot \mathbf{b}_{\perp })}{\mathbf{q}_{\perp }^2+m^{2}}=\frac{1}{2\pi }K_{0}(mb_{\perp }), \end{aligned}$$

we write

27 $$\begin{aligned} E^{II}=-\frac{\sigma _{1}\sigma _{2}}{2\pi \sqrt{1-\mathbf{v}_{\perp }^{2}}}\left( \frac{v^{3}v^{0}}{1+v^{2}}\right) \lim _{m\rightarrow 0}[K_{0}(mb_{\perp })], \end{aligned}$$

where $K_{0}(mb_{\perp })$ stands for the K-Bessel function and $b_{\perp }=\left| \mathbf{b}_{\perp }\right|$.

Now, we use the fact that [34] $K_{0}(mb_{\perp })\mathop {\rightarrow }\limits ^{m\rightarrow 0}-\ln \left( mb_{\perp }/2\right) -\gamma$, where $\gamma$ is the Euler constant, in order to handle the expression (27); thus

28 $$\begin{aligned} E^{II}&= \frac{\sigma _{1}\sigma _{2}}{2\pi \sqrt{1-\mathbf{v}_{\perp }^{2}}}\left( \frac{v^{3}v^{0}}{1+v^{2}}\right) \lim _{m\rightarrow 0}\left[ \ln \left( \frac{mb_{\perp }}{2}\right) +\gamma \right] \nonumber \\&= \frac{\sigma _{1}\sigma _{2}}{2\pi \sqrt{1-\mathbf{v}_{\perp }^{2}}}\left( \frac{v^{3}v^{0}}{1+v^{2}}\right) \lim _{m\rightarrow 0}\biggl [\ln \biggl (\frac{mb_{\perp }}{2}\biggr )+\gamma \nonumber \\&+\ln (ma_{0})-\ln (ma_{0})\biggr ]\nonumber \\&= \frac{\sigma _{1}\sigma _{2}}{2\pi \sqrt{1-\mathbf{v}_{\perp }^{2}}}\left( \frac{v^{3}v^{0}}{1+v^{2}}\right) \nonumber \\&\times \biggl [\ln \left( \frac{b_{\perp }}{a_{0}}\right) \left[ \gamma -\ln 2+\lim _{m\rightarrow 0}\ln (ma_{0})\right] \biggr ]. \end{aligned}$$

Here, in the third line, we added and subtracted the quantity $\ln (ma_{0})$, where $a_{0}$ is an arbitrary constant having the dimension of length. We can neglect the last line of Eq. (28) since it does not depend on the distance $a_{\perp }$ and, therefore, does not contribute to the force between line current and the point charge. Defining ${\hat{I}}$ as the unit vector along the straight line current and noticing that $v^{3}$ is the projection of the vector $\mathbf{v}$ along the straight line current, one can identify $v^{3}=\mathbf{v}\cdot {\hat{I}}$. Therefore, the energy can be written as

29 $$\begin{aligned} E^{II}=\frac{\sigma _{1}\sigma _{2}}{2\pi }\frac{\mathbf{v}\cdot {\hat{I}}}{\sqrt{1-\mathbf{v}_{\perp }^{2}}}\left( \frac{v^{0}}{1+v^{2}}\right) \ln \left( \frac{b_{\perp }}{a_{0}}\right) , \end{aligned}$$

where

30 $$\begin{aligned} b_{\perp }=\sqrt{\mathbf{a}_{\perp }^{2}+\frac{(\mathbf{v}_{\perp }\cdot \mathbf{a}_{\perp })^{2}}{1-\mathbf{v}_{\perp }^{2}}}. \end{aligned}$$

At this point, some comments are in order. The interaction energy (29) is an effect due solely to the Lorentz-symmetry breaking and has no counterpart in Maxwell theory, where a point-like charge does not interact with a steady line current. This fact can be seen by noticing that in the limit $v^{\mu }=0$, the energy (29) vanishes. The energy (29) is proportional to the electric charge $\sigma _{2}$ as well as to the projection of the Lorentz-symmetry breaking vector $\mathbf{v}$ along the current line, which is proportional to $\sigma _{1}\mathbf{v}\cdot {\hat{I}}$. If the current line flows perpendicular to $\mathbf{v}$, there is no interaction. If $v^{\mu }$ is a space-like vector and $v^{0}=0$ the energy, Eq. (29) vanishes too. The spatial dependence of the force between the charge and the steady current falls off as the distance increases with an inverse law, in a first order approximation. The exact expression for the force is

31 $$\begin{aligned}&\mathbf{F}^{II}=-\nabla _{\mathbf{a}_{\perp }}E^{II}=\frac{\sigma _{1}\sigma _{2}}{2\pi }\frac{\mathbf{v}\cdot {\hat{I}}}{\sqrt{1-\mathbf{v}_{\perp }^{2}}}\left( \frac{v^{0}}{1+v^{2}}\right) \nonumber \\&\quad \times \Biggl [\mathbf{a}_{\perp }^{2}+\frac{(\mathbf{v}_{\perp }\cdot \mathbf{a}_{\perp })^{2}}{1-\mathbf{v}_{\perp }^{2}}\Biggr ]^{-1}\Biggl [\mathbf{a}_{\perp }+\frac{(\mathbf{v}_{\perp }\cdot \mathbf{a}_{\perp })}{1-\mathbf{v}_{\perp }^{2}}\mathbf{v}_{\perp }\Biggr ]. \end{aligned}$$

As a final comment, we point out that if we fix the point charge, the energy (29) leads to a torque on the steady current, which can be calculated. We denote by $\theta$ the angle between $\mathbf{v}_{\perp }$ and $\mathbf{a}_{\perp }$, and we proceed as follows:

32 $$\begin{aligned} \tau ^{II}&= -\frac{\partial E^{II}}{\partial \theta }\nonumber \\&= -\frac{\sigma _{1}\sigma _{2}}{4\pi }\frac{\mathbf{v}\cdot {\hat{I}}}{\sqrt{1-\mathbf{v}_{\perp }^{2}}}\left( \frac{v^{0}}{1+v^{2}}\right) \frac{\mathbf{v}_{\perp }^{2}\sin (2\theta )}{1-\mathbf{v}_{\perp }^{2}\sin ^{2}(\theta )}. \end{aligned}$$

If $\theta =0,\pi /2,\pi$ the torque (32) vanishes.

## Dirac strings

In this section we consider the system composed by a point-like charge placed at position $\mathbf{a}$ and a Dirac string. This system is described by the external source,

33 $$\begin{aligned} J^{\mu ,III}(x)=J_{(D)}^{\mu }\left( x\right) +\sigma \eta ^{0\mu }\delta ^{3}(\mathbf{x}-\mathbf{a}), \end{aligned}$$

where $J_{(D)}^{\mu }\left( x\right)$ stands for the external field source produced by the Dirac string and the second term on the right hand side for the source produced by the point-like charge.

Now, we choose a coordinate system where the Dirac string lies along the $z$-axis with internal magnetic flux $\varPhi$. Its corresponding source is given by [35, 36]

34 $$\begin{aligned} J_{(D)}^{\mu }(\mathbf{x})=i\varPhi (2\pi )^{2}\int \frac{\mathrm{d}^{4}p}{(2\pi )^{4}}\delta (p^{0})\delta (p^{3})\varepsilon ^{0\mu }_{\ \ \nu 3}\ p^{\nu }e^{-ipx}.\nonumber \\ \end{aligned}$$

If $\varPhi >0$ we have the internal magnetic field along $\hat{z}$. For $\varPhi <0$, the internal magnetic field points in the opposite direction.

From now on, in this section, the sub-index $\perp$ means that we are taking just the components of a given vector perpendicular to the string. For instance, $\mathbf{p}_{\perp }=(p_{x},p_{y},0)$ is the momentum perpendicular to the string.

Substituting Eq. (34) into Eq. (33) and employing the result with Eq. (5), using the Fourier representation (3) for the propagator $D^{\mu \nu }(x,y)$, discarding self-interacting terms, which do not contribute to the force between the string and the charge (the self-interacting terms are proportional to $\sigma ^2$ or $\varPhi ^2$), and following similar steps employed in the previous sections, we show that

35 $$\begin{aligned} E^{III}&= -i\sigma \varPhi \left( \frac{v^{0}}{1+v^{2}}\right) \int \frac{\mathrm{d}^{2}\mathbf{p}_{\perp }}{(2\pi )^{2}}\frac{\left[ \hat{z}\cdot \left( \mathbf{p}_{\perp }\times \mathbf{v}_{\perp }\right) \right] }{[\mathbf{p}_{\perp }^2-(\mathbf{v}_{\perp }\cdot \mathbf{p}_{\perp })^{2}]}\nonumber \\&\times \exp \left( i\mathbf{p}_{\perp }\cdot \mathbf{a}_{\perp }\right) . \end{aligned}$$

Now, we make a change of the integration variables (8), (9), (10), (11), and (12) just for the $\perp$ coordinates in order to rewrite the expression (35) in the form

36 $$\begin{aligned} E^{III}&= -\frac{\sigma \varPhi }{\sqrt{1-\mathbf{v}^{2}_{\perp }}}\left( \frac{v^{0}}{1+v^{2}}\right) \left[ \hat{z}\cdot \left( \mathbf{\nabla }_{\mathbf{b}_{\perp }}\times \mathbf{v}_{\perp }\right) \right] \nonumber \\&\times \lim _{m\rightarrow 0}\int \frac{\mathrm{d}^{2}\mathbf{q}_{\perp }}{(2\pi )^{2}}\frac{\exp \left( i\mathbf{q}_{\perp }\cdot \mathbf{b}_{\perp }\right) }{\mathbf{q}^{2}_{\perp }+m^{2}}, \end{aligned}$$

where we inserted a regulator parameter $m$ with the dimension of a mass in order to eliminate divergences and we defined the differential operator

37 $$\begin{aligned} \mathbf{\nabla }_{\mathbf{b}_{\perp }}=\left( \frac{\partial }{\partial b^{1}},\frac{\partial }{\partial b^{2}}\right) . \end{aligned}$$

Substituting Eq. (26) in Eq. (36) and performing some simple calculations we show that

38 $$\begin{aligned} E^{III}&= \lim _ {m\rightarrow 0}\frac{\sigma \varPhi }{2\pi \sqrt{1-\mathbf{v}^{2}_{\perp }}}\left( \frac{v^{0}}{1+v^{2}}\right) \left[ \frac{mK_{1}\left( m\mathbf{b}_{\perp }\right) }{|\mathbf{b}_{\perp }|}\right] \nonumber \\&\times \left[ \hat{z}\cdot \left( \mathbf{b}_{\perp }\times \mathbf{v}_{\perp }\right) \right] . \end{aligned}$$

We now take the limit $m\rightarrow 0$, and we use definition (11) and the fact that $\varPhi {\hat{z}}=|\varPhi |{\hat{B}}_{\mathrm{int}}$, where ${\hat{B}}_{\mathrm{int}}$ is the unit vector pointing in the internal magnetic field direction. This leads to

39 $$\begin{aligned} E^{III}&= \frac{\sigma |\varPhi |}{2\pi \sqrt{1-\mathbf{v}^{2}_{\perp }}}\left( \frac{v^{0}}{1+v^{2}}\right) \left[ \mathbf{a}_{\perp }^{2}+\frac{(\mathbf{v}_{\perp }\cdot \mathbf{a}_{\perp })^{2}}{1-\mathbf{v}_{\perp }^{2}}\right] ^{-1}\nonumber \\&\times \left[ {\hat{B}}_{\mathrm{int}}\cdot \left( \mathbf{a}_{\perp }\times \mathbf{v}_{\perp }\right) \right] . \end{aligned}$$

We notice that Eq. (39) is also an exclusive effect due to the Lorentz-symmetry breaking. In the limit $v^{\mu }\rightarrow 0$ the energy (39) vanishes.

The energy (39) leads to a force between the Dirac string and the charge as well as a torque on the string, by fixing the charge position.

The fourth example is given by two parallel Dirac strings placed a distance $a$ apart. We take a coordinate system where the first string lies along the $z$-axis, with internal magnetic flux $\varPhi _{1}$ and where the second one, lying along the line $\mathbf{a}=(a^{1},a^{2})$ has $\varPhi _{2}$. The corresponding external source is given by

40 $$\begin{aligned} J_{\mu }^{IV}\left( \mathbf{x}\right) =J_{\mu (D,1)}\left( \mathbf{x}\right) +J_{\mu (D,2)}\left( \mathbf{x}\right) , \end{aligned}$$

where $J_{(D,1)}^{\mu }\left( \mathbf{x}\right)$ has the same form as Eq. (34), with $\varPhi$ replaced by $\varPhi _{1}$, and

41 $$\begin{aligned} J_{(D,2)}^{\mu }\left( \mathbf{x}\right) \!=\!i\varPhi _{2}\int \frac{\mathrm{d}^{4}p}{(2\pi )^{2}}\delta (p^{0})\delta (p^{3})\varepsilon ^{0\mu }_{\ \ \nu 3}\ p^{\nu }e^{-ipx}e^{-i\mathbf{p}_{\perp }\cdot \mathbf{a}}.\nonumber \\ \end{aligned}$$

Proceeding as in the previous cases, we show that the interaction energy between the two Dirac strings is given by

42 $$\begin{aligned}&E^{IV}=\frac{L{\varPhi }_{1}{\varPhi }_{2}}{\sqrt{1-\mathbf{v}^{2}_{\perp }}}\Biggl [-\Biggl (1+\frac{\mathbf{v}_{\perp }^{2}}{1+v^{2}}\Biggr )\int \frac{\mathrm{d}^{2}\mathbf{q}_{\perp }}{(2\pi )^{2}}e^{i\mathbf{q}_{\perp }\cdot \mathbf{b}_{\perp }}\nonumber \\&\quad +\Biggl (\frac{1}{1+v^{2}}-\frac{1}{1-\mathbf{v}_{\perp }^{2}}\Biggr )\int \frac{\mathrm{d}^{2}\mathbf{q}_{\perp }}{(2\pi )^{2}}\frac{\left( \mathbf{v}_{\perp }\cdot \mathbf{q}_{\perp }\right) ^{2}}{\mathbf{q}^{2}_{\perp }}e^{i\mathbf{q}_{\perp }\cdot \mathbf{b}_{\perp }}\Biggr ],\nonumber \\ \end{aligned}$$

where we identified the Dirac string length $L=\int \mathrm{d}x^{3}$.

The first term inside the brackets of Eq. (42) is the Dirac delta function $\delta ^{2}(\mathbf{b}_{\perp })$ and provided that $\mathbf{a}_{\perp }$ is nonzero (and therefore $\mathbf{b}_{\perp }$ is also nonzero), this term vanishes. Using definition (37) and inserting a mass parameter, as we have in Eq. (25), we find

43 $$\begin{aligned}&E^{IV}=-\frac{L{\varPhi }_{1}{\varPhi }_{2}}{\sqrt{1-\mathbf{v}^{2}_{\perp }}}\Biggl (\frac{1}{1+v^{2}}-\frac{1}{1-\mathbf{v}_{\perp }^{2}}\Biggr )\nonumber \\&\quad \times \left( \mathbf{v}_{\perp }\cdot \mathbf{\nabla }_{\mathbf{b}_{\perp }}\right) ^{2}\lim _{m\rightarrow 0}\int \frac{\mathrm{d}^{2}\mathbf{q}_{\perp }}{(2\pi )^{2}}\frac{\exp \left( i\mathbf{q}_{\perp }\cdot \mathbf{b}_{\perp }\right) }{\mathbf{q}^{2}_{\perp }+m^{2}}. \end{aligned}$$

Using the result (26), taking the limit $m\rightarrow 0$, acting with the differential operators, and using Eq. (30), we have

44 $$\begin{aligned} \mathcal{E}^{IV}&= \frac{E^{IV}}{L}=\frac{1}{2\pi }\frac{{\varPhi }_{1}{\varPhi }_{2}}{\sqrt{1-\mathbf{v}^{2}_{\perp }}}\Biggl (\frac{1}{1+v^{2}}-\frac{1}{1-\mathbf{v}_{\perp }^{2}}\Biggr )\nonumber \\&\times \left( \mathbf{a}_{\perp }^{2}+\frac{(\mathbf{v}_{\perp }\cdot \mathbf{a}_{\perp })^{2}}{1-\mathbf{v}_{\perp }^{2}}\right) ^{-2}\nonumber \\&\times \Biggl [\mathbf{v}_{\perp }^{2}\mathbf{a}_{\perp }^{2}-\Biggr (\frac{2-\mathbf{v}_{\perp }^{2}}{1-\mathbf{v}_{\perp }^{2}}\Biggr )(\mathbf{v}_{\perp }\cdot \mathbf{a}_{\perp })^{2}\Biggr ], \end{aligned}$$

where we defined the energy per unit of string length $\mathcal{E}$.

It can be seen that the energy given above vanishes in the limit $v^{\mu }=0$, where we do not have Lorentz-symmetry breaking. The interaction energy (44) is an effect due solely to the Lorentz-symmetry breaking and has no counterpart in Maxwell theory.

The fifth and last model we consider is given by a Dirac string and a steady line current, the two parallel to each other. The corresponding external source is

45 $$\begin{aligned} J^{V}_{\mu }(\mathbf{x})=\sigma \eta ^{3}_{\ \mu }\delta ^{2}\left( \mathbf{x}_{\perp }-\mathbf{A}\right) +J_{(D)}^{\mu }\left( \mathbf{x}\right) \end{aligned}$$

where $J_{(D)}^{\mu }\left( \mathbf{x}\right)$ is given by Eq. (34).

Following similar steps as previously we have

46 $$\begin{aligned} \mathcal{E}^{V}&= \frac{E^{V}}{L}=\frac{\sigma |\varPhi |}{2\pi \sqrt{1-\mathbf{v}^{2}_{\perp }}}\left( \frac{\mathbf{v}\cdot {\hat{I}}}{1+v^{2}}\right) \nonumber \\&\times \left[ \mathbf{a}_{\perp }^{2}+\frac{(\mathbf{v}_{\perp }\cdot \mathbf{a}_{\perp })^{2}}{1-\mathbf{v}_{\perp }^{2}}\right] ^{-1}\left[ {\hat{B}}_{\mathrm{int}}\cdot \left( \mathbf{a}_{\perp }\times \mathbf{v}_{\perp }\right) \right] . \end{aligned}$$

We note that Eq. (46) also represents an exclusive effect of Lorentz-symmetry breaking.

## Conclusions and final remarks

In this paper, the interactions between stationary electromagnetic field sources were investigated in a Lorentz-symmetry breaking scenario in $(3+1)$ dimensions. The Lorentz-symmetry breaking parameter has been chosen to be a vector one, in such a way that all the results obtained are exact. We observed effects with no counterpart in Maxwell electrodynamics and that have not been explored in the literature up to now. We showed the emergence of a spontaneous torque on a classical electromagnetic dipole and an interaction between a steady straight line current and a point-like charge. Both phenomena are due solely to the Lorentz-symmetry breaking.

We also investigated some phenomena due to the presence of a Dirac string. We showed that this string can interact with a point charge as well as with a straight line steady current. We also studied the interaction between two parallel Dirac strings. These results suggest that in models which exhibit explicit Lorentz-symmetry breaking the Aharonov–Bohm effect can be modified in comparison with the results predicted by Maxwell electrodynamics. This subject deserves more investigations not only in the context of the model considered in this paper, but also for other kinds of models with Lorentz-symmetry breaking.

Finally, we would like to comment that taking into account the spin of the sources would be a natural follow-up of the investigation we have pursued in the present paper. If we consider the situation where the charge carriers (our sources) may have spin 1/2 or 1 (here our sources are spinless), the expressions for the interacting energies and forces worked out here shall display a spin dependence; not only a spin–spin interaction between the sources appears, but also a non-trivial coupling between the source’s spin and the background vector shows up. Then the spin dependence of the interaction energy and force may disclose reveal a number of peculiar effects with no counterpart in the usual Maxwellian electrodynamics. We shall soon be reporting on the results of these studies [37].
